# Lady Hale: spider woman

**DOI:** 10.1192/bjb.2022.28

**Published:** 2022-08

**Authors:** Claire McKenna

**Figure:**
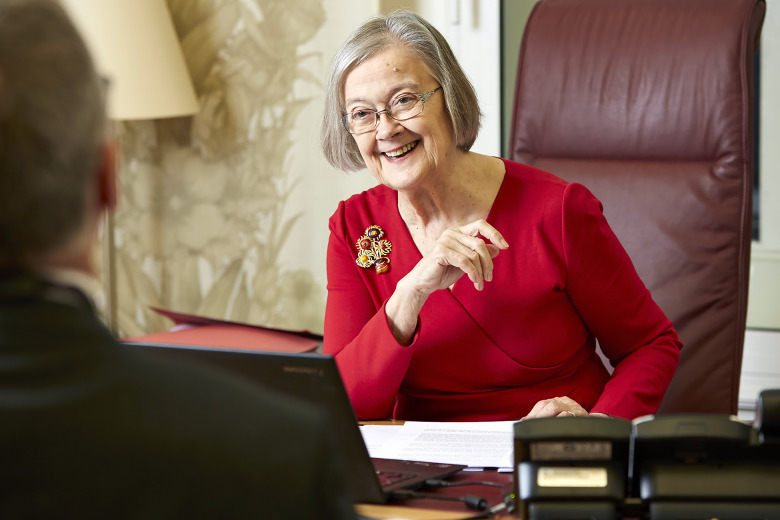

Lady Hale is *delightful*. She is warm and friendly, in a straightforward kind of way. She has been described in other interviews as someone men were ‘afraid of’ and a woman who ‘won't indulge in mushy stuff’ one wonders if there is more than a weak undertow of sexism in the depictions of the first female president of the Supreme Court, who became a household name when she calmly delivered the ‘unlawful, void and of no-effect’ judgment on Boris Johnson's 2019 attempt at proroguing Parliament. But then she has been fighting stereotypes and dashing people's preconceptions of her all her life.

‘I have indeed, on the whole, forged ahead with what I wanted to do or what other people wanted me to do, irrespective of obstacles, criticisms, denigration, sexist remarks or whatever was going on. Sometimes I knew what they were. Most of the time I didn't know what they were. But in any event, it wouldn't have stopped me doing whatever it was I thought I ought to be doing.’

Nicknamed ‘the Beyoncé of the legal world’ for her popularity with law students, her back catalogue will perhaps be most familiar to psychiatrists in the form of the ‘Cheshire West’ case,^1^ which set the legal ‘acid test’ for deprivation of liberty and where Hale coined the memorable phrase ‘a gilded cage is still a cage’.

Retired as a judge since January 2020, Hale has said that she felt it necessary throughout her career to speak up for the most disadvantaged in our society, particularly children and those with mental health problems and a learning disability.

## Coming full-circle

Hale's enduring interest in mental health law was piqued when she taught law to social workers and psychiatrists, at Manchester University, in what became an almost 20 year career in academia. She asks me to remember that she's been retired for 2 years and might not be up to date. This is surprisingly humble, given that in 1975 she wrote the first ever textbook for mental health practitioners on mental health law in the UK, the sixth edition of which is in print with her as lead author.

A complete sea change has occurred in attitudes to protecting those who can't consent to their care and treatment in Hale's lifetime. ‘The Mental Health Act (1959) had been passed with a view to getting the law, the lawyers and courts out of mental health and leaving it to the professionals to decide what should happen to people in mental health difficulties’, she says. In the intervening period, however, patients, their families and lawyers have highlighted the need for safeguards against interventions that are an invasion of people's rights to autonomy, bodily integrity and liberty. ‘I think for a long time we did assume that people were acting for the best and that we didn't really need safeguards other than the normal criminal law against ill treatment and neglect.’

She observes that there has always been a tension between professionals who are trying to do their best for patients and the need for checks on what they are doing, but professionals haven't always been sensitive to it.

‘I think the psychiatrists got more and more conscious of their patients’ rights as time went on. When I first started, they were, on the whole, a little bit dismissive of this. Whereas as time went on, I found that on the whole, the psychiatrists were very, very sensitive to what they were doing in this respect and very thoughtful about it.’

The Bournewood judgment^2^ was a seminal case, beginning the shift towards the need for safeguards to deprive someone of their liberty, including for people with mental disabilities who can't give informed consent. Hale acknowledges, however, that we may now be in a situation which is confusing for practitioners, particularly in the interface between the Mental Health and Mental Capacity Acts.

‘It does inevitably mean that there are procedures which it might be easier to do without. I agree that there have to be safeguards in both types of situation. But whether they have to be as complicated as they are, whether one could unite the Mental Health Act and the Mental Capacity Act into a single system that operated in all kinds of eventualities that might arise for people with all kinds of diagnoses … I think that would be the right thing. We'd be back to the Mental Health Act of 1959, of course, which was trying to do the same.’

## Concerns about the Mental Health Act

Lady Hale shares the well-rehearsed concerns about the Mental Health Act 1983 (MHA) described in Professor Sir Simon Wessley's 2018 review, including that it is likely more people are sectioned than necessary.

‘It [the MHA] is still probably too authoritarian. In that if you look at the criteria for sectioning somebody, really there's very little to them, they're extremely broad. And they don't make a serious attempt to distinguish between serious mental illness and other forms of mental health problem … And basically, unless whoever's operating them imposes their own restricting criteria, it would be capable of being used in a far wider set of circumstances than really can be justifiable in principle, it seems to me. I think that's my major concern about it.’

She is also concerned that a shortage of in-patient beds means that professionals are more likely to use formal procedures to obtain one. ‘It shouldn't be necessary to section somebody in order to get a bed if somebody needs a bed.’

Northern Ireland's very recent introduction of ‘fusion legislation’ is something Hale watches with keen interest and is ‘hoping it works out well’. The MCA (NI) 2016 is the first legislation of its kind, aiming to provide a framework for the care and treatment of people who lack capacity to consent, across all areas of health and social care. Hale sees it as a potential way to resolve some of the confusion and complications of the English system.

‘I think in principle the justification for doing things to and with people who lack the capacity or who don't consent to it, is that they lack the capacity to consent to it. That's the best philosophical justification for interfering with their autonomy in that way. Of course, it does depend on what you mean by lack of capacity. But I think that it's possible to devise a definition of lack of capacity which would cater for the major mental illnesses as well, because of the way in which they interfere with the mental decision-making process. And so my own view is that's the right way to go.

‘I've got some of the way towards persuading the Mental Health Act review here that that might in the long run be the right way to go. But I think they're waiting to see how things work out in Northern Ireland before they adopt something like that.’

The discussion on whether to remove learning disability and autism from the Mental Health Act is a particularly fraught one, but Hale suggests that we may be asking the wrong question. An approach based on a test of capacity would make the condition for detention – whether mental illness or mental disability – secondary, she says. ‘They ought all to be in a single, simple, coherent system. But that's the lawyer in me, you see, that likes it to be principled and to get away from the notion that this is a stigmatising thing, as opposed to a necessary safeguard for people who, in their own best interests, have to have their liberty curtailed.’

She concedes that judges themselves may have inadvertently contributed to a culture of ‘blame, shame and fear’, which predisposes professionals to detain people in hospital rather than care for those with behaviours which may be high risk in the community.

‘All judges have to deal with actions for compensation where things have gone wrong. And those actions tend to depend upon somebody being at fault, so necessarily talk about assigning blame. And from time to time, I think judges probably use far too judgmental language when they are assigning blame, when in fact, it ought to be possible to say, well, this shouldn't have happened and there was a failure to take reasonable care here, without being particularly condemnatory of the professionals who were involved in that.’

Like many, she also believes that there are too many people with mental health problems or a learning disability in prison, and ‘that will continue to be a problem until there is more political pressure in favour of providing proper resources in the community’.

## The least detrimental alternative

Doctors and lawyers have a certain affinity with one another, Hale believes, particularly lawyers working in family law, where she started her judging career. The ‘touchy feely areas’ of welfare law and equality law generally exerted a pull on her throughout her career. As a judge, she feels one must ‘understand the client or the parties who are appearing in front of you, get some sort of empathy for them, where they're coming from, but at the same time, maintain the distance that you require in order to stand back and make the right decision’. And, like psychiatrists, family lawyers rarely deal in black and white, only shades of grey. ‘As a family lawyer, the role is to make the future of this family better than it otherwise might be. Well, people sometimes talk about the least detrimental alternative.’

She allows that hasn't always been easy. As doctors and judges, we are bound by the constraints of the systems we work in. She points out that for doctors and social workers working with children and families, resource constraints mean ‘the community pressure is so frequently towards removal and incarceration. That's a perpetual problem for which the solutions are on the whole, political rather than legal’.

Has she ever felt conflicted between an outcome that was correct in law and what she felt to be morally right?

‘As a family division judge, the most difficult area of jurisprudence was to do with child abduction because under the Hague Convention on child abduction, which is part of UK law, you have to send a child who has been abducted from their home country back home in most circumstances … But there are plenty of cases where you know that they're going to be better off here than they would be back in their home country. Those were the hardest cases, where the law obliged you do something that you didn't want to do because of the children involved.’

## Doublethink

The poverty of provision for children with complex needs in the UK, including those with learning disabilities and those who can't remain at home for one reason or another, is the area of children's rights Hale is most concerned about.

‘A particular area of concern in England is that social work has become so pressed for resources to help families to stay together and look after their children safely, and so risk averse because of the criticism that they're subject to if abuse is missed, that a great many children are being removed from their families who almost certainly shouldn't be removed from their families. With better help and support they could stay together. And that's a really serious worry. I think we've got escalating numbers of children in care and increasing difficulties in finding the right ways of caring for them, which aren't going to do more harm than good.’

In 1984, Hale became the first woman and youngest person to be appointed to the Law Commission for England and Wales, which promotes reform of the law. Among her proudest achievements there were her leading roles in the development of the Children Act 1989 (or ‘Brenda's weird child law’, as it was referred to by her colleagues!), as well as what became the Mental Capacity Act in 2005. Concern for the welfare of children is a seam running through her career. She points out the contradictions inherent in the legislation relating to them, epitomised by the UK having Europe's lowest age of criminal responsibility at age 10.

‘There's a lot of doublethink about children. We're quite reluctant to afford them autonomy and decision making capacities except when it comes to their offending against the criminal law, when we treat them as mini-adults. It seems extraordinary to me, the mismatch between our view of them in family law and in civil law and our view of them in criminal law. They ought to be consistent.’

## Wicked problems

Hale is often compared with fellow Supreme Court trailblazer Ruth Bader Ginsberg, but unlike her US counterpart's famous ‘dissent collars’, Hale does not intend her choice of brooches to communicate a hidden message. My attempt to prise layers of metaphor from the title of her 2021 memoir *Spider Woman: A Life* prove fruitless.

‘I'm afraid the reference was to the brooch I was wearing when I delivered the judgment of the Supreme Court in the case about the prorogation of Parliament. And I happened to be wearing a spider brooch on my dress, and that got more publicity, I think, than the actual judgment itself did. So I thought the thing to do is to embrace that and capitalise on it really.’

In her memoir, Hale comes across as methodical and direct in her approach to life, and during our hour-long conversation over Zoom, she is resistant to this psychiatrist's probing for hidden meaning.

But it would have been remiss of me not to use my audience with one of the world's finest legal minds to try to resolve some of the legal dilemmas relevant to my own sphere of practice as a child and adolescent psychiatrist in intellectual disability. How, for example, should Northern Ireland's new mental capacity legislation deal with the under-16 s?

‘I think the under-16 s are a problem everywhere. We've had quite a lot of litigation here, but not only for under-16 s, 16 and 17 year olds as well [a reference to the 2019 Re: D case,^3^ where she delivered the main judgment, finding that parents could not consent to deprive a 16 or 17 year old of their liberty]. There are really tricky questions about whether you have a separate regime for them and what the regime should be and to what extent should it recognise children's autonomy. And I don't have any simple answers to that at all.’

Oh well. I try again. What about the use of mental health legislation more generally for under-16 s. Does she support my use of detention for young in-patients subject to an extremely restrictive programme of care to which they cannot consent?

‘For a long, long time I've been worried that the anxiety to spare any patient, but particularly a child patient, what is seen as the stigma of having been the subject to formal processes actually, of course, deprives those people of the protection which the formal processes are designed to give them. And if we think that anybody deserves protection against what I am sure is well-meaning but misguided attempts to help them or secure them, the need for protection is just as great with young people as it is with older people. So I think I've always thought that was the right position in principle. But of course, in practice, you want your safeguards to be not too bureaucratic and more readily operable and not too time-consuming, as long as there are some safeguards. And so I think I support what your practice is.’

Success! When I joke that it's good to have a former Supreme Court justice supporting my position, she reminds me in her lawyerly way that of course she has only heard my side of the story.

## Bending towards justice?

Now in retirement, Hale has set herself the ambitious task of writing a book to educate the lay person on what the law can do *for* people as opposed to against them, which, she says, is ‘a very much more difficult job than writing about oneself. If I could pull off the book that I'm struggling with, I think that would be worth achieving because it would be an attempt to put over to everybody in the country how important the law is for them’.

Paraphrasing Martin Luther King's famous adage, I ask her if we are as a society ‘bending towards justice’ for our most vulnerable – children and those with mental illness and a learning disability. I'm not so sure sometimes. Is she?

‘For most of my professional life, I think one could look at the law and think that it was improving in many respects. But we have now a situation where the justice system is under incredible strain, having been starved of resources for more than a decade now. And the people who use the justice system are also under incredible strain, again because of the cutbacks in publicly funded legal services of all sorts. So we have got a big problem there, which is getting worse.

‘There are opportunities, but the trouble with the opportunities is that they cost money. I would love us to be able to digitise legal procedures, so that all you would have to do is go online and tell the court what the problem was and what you wanted and be guided through the legal processes and out the other end, just as if you were taxing your car or something like that … But of course, it costs a lot of money. So in the meantime, I think I'm more worried than I am optimistic.’

## About the author

**Claire McKenna** is a consultant child and adolescent psychiatrist in intellectual disability at The Iveagh Centre, Belfast Health and Social Care Trust, Belfast, UK.
